# Psychometric testing of the Older Patients in Acute Care Survey (OPACS) in Australian final year nursing students

**DOI:** 10.1002/nop2.238

**Published:** 2019-02-06

**Authors:** Helen Venables, Yvonne Wells, Deirdre Fetherstonhaugh

**Affiliations:** ^1^ College of Science, Health and Engineering La Trobe University Melbourne Vic. Australia; ^2^ Lincoln Centre for Research on Aging La Trobe University Melbourne Vic. Australia; ^3^ Australian Centre for Evidence Based Aged Care La Trobe University Melbourne Vic. Australia

**Keywords:** attitude, knowledge, older patients, OPACS, student nurses

## Abstract

**Aim:**

To assess the internal reliability and validity (content and criterion) of the Older Patients in Acute Care Survey (OPACS) as a measure of nurses’ knowledge, attitudes and practices regarding care of older hospitalized patients in Australia.

**Design:**

Cross‐sectional survey.

**Method:**

A convenience sample of final year nursing students at an Australian university in October 2014 completed the OPACS (*N = *191). Internal reliability was assessed using Cronbach's alpha, content validity using exploratory factor analysis and criterion validity using correlations between the OPACS and Palmore's Facts on Aging Quiz and the Caring Efficacy Scale.

**Results:**

Despite good internal reliability on both OPACS subscales, exploratory factor analysis of the 36 items representing behaviours and the 50 items on knowledge and attitudes failed to load strongly on their corresponding factors. Analyses of criterion validity suggested the OPACS scales are measures of attitude.

## 1 INTRODUCTION

Nurses’ knowledge, skills and attitudes are critical in determining how physical, psychological and emotional care are provided to older people, who are the largest patient group in most hospital environments (Rana & Upton, 2013). However, evidence from multiple studies in a diverse range of countries demonstrates that many nurses hold negative, or only marginally positive, attitudes about working with older patients and engage in undesirable practices as a result, which contributes to a failure to provide older patients with appropriate care (Dahlke, Phinney, Hall, Rodney, & Baumbusch, 2015; Rush, Hickey, Epp, & Janke, 2017; Wilson et al., 2017). Nurses’ behaviour is influenced by their attitudes, and negative attitudes can adversely affect the quality of care provided (Kydd et al., 2014).

Hospitalization can be a life‐changing event, even for a previously healthy older person, and can be associated with the onset of a new disability (Boltz, Resnick, Capezuti, & Shuluk, 2014; Dicks, Chaplin, & Hood, 2013). Older patients generally present to acute care with more complex chronic health issues than those who are younger (Lakhan et al., 2011), and the experience of hospitalization places older patients at risk of experiencing poor outcomes, including functional decline. For older patients, any loss of function has serious ramifications as it can result in iatrogenic infections, pressure injuries, falls, non‐elective rehospitalization and increased risk of mortality (Boltz, Resnick, Capezuti, Shuluk, & Secic, 2012).

Nurses’ attitudes towards older patients are established early in their professional identity formation. Student nurses have been shown to demonstrate more negative attitudes towards older patients than those younger and having man**y** interactions with older patients may increase prejudice (Kydd et al., 2014; van Leeuwen, Oosterhuis, & Ruyter, 2016). These attitudes may reflect those of a youth‐oriented society (Chrisler, Barney, & Palatino, 2016; Heise, Johnsen, Himes, & Wing, 2012) or may be promoted by Bachelor of Nursing (BN) programs that focus on a biomedical model of ageing and links extreme illness and death with ageing (Henderson, Xiao, Siegloff, Kelton, & Paterson, 2008). Students may enter the BN with positive or neutral attitudes towards older people, but their experiences in gerontological practical placements negatively influence their desire to work with older people as a career choice (Heise et al., 2012).

The stresses associated with the transition from university to the hospital environment are well documented in the literature and discrepancies between undergraduates’ expectations based on their education, and the actual work environment can create a sense of instability for graduate nurses (Duchscher, 2009). Hospitals practise the traditional medical model of care, focusing on the medical diagnosis of the patient and overlooking the specific functional needs of older patients (Asmus‐Szepesi et al., 2015; Buurman et al., 2011). These issues may challenge and frustrate nurses’ attempts to provide care for older patients in a hospital system that is not designed to manage their complex needs (Dahlke et al., 2015). Further, concern has been expressed that the culture of nursing has changed over the last few decades, with a decrease in nurses’ willingness and motivation to help older patients (Koskenniemi, Leino‐Kilpi, & Suhonen, 2013).

There remains a dearth of studies designed to investigate the attitudes (and the associated factors) of student nurses regarding older people (Liu, While, Norman, & Wenqin, 2012). One reason for this is the lack of suitable tools to measure ageism in nurses in the hospital sector. Courtney, Tong, and Walsh (2000) described the scarcity of literature examining nurses’ knowledge, attitudes and practices towards older hospitalized patients as “extremely disturbing” (p. 95). Kogan's Attitude towards Old People scale was at the time and continues to be commonly used (Dikken, Hoogerduijn, Lagerwey, et al., 2017; Neville & Dickie, 2014); however, the limitations of this survey are becoming increasing apparent. Social attitudes, the health care sector and nursing education have all evolved since it was developed in America in the middle of last century, and the focus of Kogan's scale is on caring for older people generally, rather than specifically hospitalized older patients (Runkawatt, Gustafsson, & Engström, 2013). Consequently, Courtney et al. (2000) developed the Older Patient in Acute Care Survey (OPACS) to address the need for a research instrument to assess nurses’ knowledge, attitudes and practices towards older hospitalized patients. This study investigated the adequacy of the OPACS among final year Australian BN students to determine its potential for use in future studies as a measure of new graduates’ preparedness to care for older patients.

### 1.1 Background

Given the need for a reliable and valid measure of nurses’ knowledge and attitudes towards older hospitalized patients, the OPACS has been the focus of studies undertaken in the Netherlands and the United States of America (USA). Studies have used both qualitative (Malmgreen, Graham, Shortridge‐Baggett, Courtney, & Walsh, 2009; van Schelven et al., 2015) and quantitative methodologies (Dikken, Hoogerduijn, Klaassen, & Schuurmans, 2017; Dikken, Hoogerduijn, Lagerwey, et al., 2017). These studies have clearly identified the importance of the cultural setting in assessing the validity of the survey, and whilst the OPACS was developed and has been used in Australia (Courtney et al., 2000; Deasey, Kable, & Jeong, 2016), no studies have examined the OPACS’ reliability or validity in the Australian context.

The aim of the original developers of the OPACS was to design and trial an instrument to measure nurses’ knowledge, attitudes and practices towards older patients. The OPACS includes 86 items relating to 13 different beliefs or practices identified in the literature and focus group interviews, believed to influence the quality of nursing care of older hospitalized patients (Courtney et al., 2000). The survey comprises: section A, a practice experience scale (36 items); and section B, a general opinion scale comprising two subscales: knowledge (21 items) and attitude (29 items; Courtney 2014, personal communication). Courtney et al. (2000) designed the items of the OPACS based on the 13 themes identified, using different numbers of items for different themes. It is not clear from Courtney et al. (2000) how the 13 themes are distributed across parts of the questionnaire. These themes have not formed the basis for any subsequent analysis and have been largely ignored.

The measure uses a five‐point Likert‐type scale of response options (ranging from 1 = *neve*r to 5 = *very frequent*). Most items are worded so that a high score indicates more negative attitudes (e.g., “I have difficulty...”; “Older patients are [negative attribute such as confused]”). Exceptions are items about involving patients in their own care, which are worded so that a high score indicates high intentions to involve patients and family members. The direction of scoring for some items is ambiguous; for example, on the item “I ask older patients whether they have incontinence problems” it is not apparent whether a high score represents a positive or a negative behaviour/attitude. Courtney et al. (2000) noted wording of items may be ambiguous and “results may not provide conclusive evidence of the identified findings” (Courtney et al., 2000, p. 100).

Courtney et al.’s (2000) study included Palmore's Facts on Aging Quiz (PFAQ; Palmore, 1977) to allow criterion‐related validity testing of the knowledge items of the OPACS. However, no results of any criterion‐related validity testing have been published by this research team.

Attempts have been made to validate the OPACS in other countries. Malmgreen et al. (2009) aimed to establish validity of the OPACS in the USA and reported high content validity (CVI) for the survey (CVI = 0.92). However, when the Dutch version of the tool was studied in the Netherlands to establish content validity, the CVI obtained was 0.62 (van Schelven et al., 2015), which is well below the benchmark of 0.80, indicating poor overall content validity. Changes to the Dutch version to represent local language and culture may have contributed to differences in results from the USA study (van Schelven et al., 2015).

The reliability and structural validity of the Dutch version of the OPACS were further tested using a quantitative study design (Dikken, Hoogerduijn, Klaassen, et al., 2017). The results of a confirmatory factor analysis demonstrated good structural validity for section A (practice) and that it measured a single construct. Good internal reliability (Cronbach's alpha = 0.82) was reported after the removal of two items. However, section B (general opinion) failed to measure a single construct, making it impossible to exclude poor items based on statistical analysis or complete further analysis (Dikken, Hoogerduijn, Klaassen, et al., 2017). Neither of the studies undertaken in the Netherlands support the use of OPACS in clinical practice or research in the Dutch context.

The most recently published study of the OPACS was undertaken in the USA to assess the structural reliability and validity of the OPACS‐US, using a quantitative study design. Despite concluding that their shortened version of the OPACS (the OPACS‐US) was a structurally valid and internally consistent measure of hospital nurses’ practice experience and general opinion towards older hospitalized patients, Dikken, Hoogerduijn, Lagerwey, et al. (2017) recommended further testing of the measure, including assessment of its criterion validity.

Table 1 summarizes published studies on psychometric testing of the OPACS. Despite evidence to support the view that nurses may leave university with established negative attitudes towards nursing older people, no attempt has been made to validate the OPACS with nursing students. Clearly, further testing of this scale is required prior to future use to ensure that the constructs it measures are clear and that it is a reliable and valid survey for use in clinical practice and research.

**Table 1:** Summary of published studies on psychometric testing of the OPACS

Authors	Country	Study design	Populations, settings	Tools	Results		
					Reliability	Face validity	Other Validity
Courtney et al. (2000)	Australia	Focus group	*N* = 16 RNs in major hospital	OPACS PFAQ		X	Content
		Survey	*N* = 5 RNs	OPACS PFAQ	Test‐retest Kappa = 0.756		
Malmgreen et al. (2009)	USA	Focus group	*N* = 4 experts	OPACS		X	CVI (Content) OPACS Section A + B 0.92 Section A: 0.97 Section B: 0.92
		Survey	*N* = 5 RNs with experience caring for older patients	OPACS (minor changes to four items)	Test–retest (Not reported)		
van Schelven et al. (2015)	The Netherlands	Survey	*N* = 10 experts in geriatric nursing	OPACS, Dutch translation (changes to 19 items)		Ratings of clarity 89% scored items as “clear”	S‐CVI (Relevance): Entire OPACS, −0.62; Practice scale = 0.61, General Opinion = 0.64 (S‐CVI should be ≥0.90). Acceptable content validity for 14/36 in section A and 22/50 in Section B
Dikken, Hoogerduijn, Klaassen, et al. (2017)	The Netherlands	Survey	*N* = 201 RNs	OPACS, Dutch translation	Alpha = 0.82		Structural using CFA OPACS Section A (practice experience): acceptable structural validity, good reliability after removal of two items. OPACS Section B (General opinion): inadequate structural validity
Dikken, Hoogerduijn, Lagerwey, et al. (2017)	USA	Survey	*N = *130 RNs	OPACS‐US Older Patients Quiz (KOP‐Q)	Section A: less six items: alpha = 0.89 Section B: less 12 items: alpha = 0.89		CFA 2 factors confirmed after removal of 6 items. Correlations with KOP‐Q: 0.35 with section A and 0.25 with Section B

This study aimed to test the internal reliability (Cronbach's alpha) and validity (content and criterion) of the OPACS (Courtney et al., 2000) as a measure of student nurses’ knowledge, attitudes and practices regarding care of older hospitalized patients in Australia. It is intended that this study will add to the international discussion regarding developing a reliable and valid measure of nurses’ knowledge, attitudes and practice towards older hospitalized patients (Dikken et al., 2016).

### 1.2 Research questions

1. What is the internal reliability of the OPACS as a measure of final year student nurses’ knowledge, attitudes and practices regarding care of older hospitalized patients in Australia?
2. What is the validity (content and criterion) of the OPACS as a measure of final year student nurses’ knowledge, attitudes and practices regarding care of older hospitalized patients in Australia?

## 2 METHOD

### 2.1 Research design

The research design is a cross‐sectional survey.

### 2.2 Sample

A convenience sample of Bachelor of Nursing (BN) students (*N = *450) at an Australian university was invited to complete the survey at the end of their final lecture in the concluding week of their BN studies (in October 2014). One hundred and ninety‐one students completed the survey (see Table 2 for participant characteristics).

**Table 2:** Demographics of participant sample

Age (range, years)	20–58 years
Age (mean, years)	26.1
Sex (%)	
Women	80.6
Men	19.4
Marital status	
Single	68.1
Partnered	29.3
Divorced	2.1
Missing	0.5
Country of birth (%)	
Australia	62.3
Philippines	11.0
China	8.4
Nepal	3.7
Other English‐speaking countries	3.1
Other non‐English‐speaking countries	11.5

### 2.3 Survey

The paper‐based self‐report survey comprised the OPACS (Courtney et al., 2000) as well as the True/False/Don't know version of Palmore's Facts on Aging Quiz (PFAQ; Palmore, 1977) and the Caring Efficacy Scale (CES; Coates, 1997). The PFAQ and the CES were included to enable criterion‐related validity testing of the knowledge and the attitude items of the OPACS, respectively.

The PFAQ is a widely used reliable and valid measure of knowledge about ageing (Baumbusch, Dahlke, & Phinney, 2012), comprising 25 items, with response options *True*, *False* or *Don't Know* (Palmore, 1977). It is a short test which relies on accurate knowledge of older people to assess ageist attitudes (Pachana, Helmes, & Gudgeon, 2013). PFAQ is scored by summing the number of correct responses: items with even numbers are *True *and items with odd numbers are *False, *with total scores ranging from 0–25. Higher scores reflect greater knowledge about ageing (Wang et al., 2010).

The CES has been found to be a reliable and valid measure of nurses’ confidence regarding their ability develop caring relationships with patients (Reid, Courtney, Anderson, & Hurst, 2015). It is a self‐report 30‐item, 6‐point (strongly disagree −3 to strongly agree +3) Likert‐type scale and contains 23 positively worded and seven negatively worded items.

### 2.4 Ethical considerations

Ethics approval was provided by the Queensland University of Technology Human Research Ethics Committee (approval number 1400000697). All participants were provided with a Participant Information Sheet explaining the aims of the study. Participation was voluntary and anonymous.

### 2.5 Data analysis

Data analysis was undertaken using IBM SPSS version 22 on the 191 complete responses. Ambiguous items in the OPACS were scored in the direction consistent with most OPACS items, so that a high score indicated more negative attitudes.

Internal reliability was assessed for each of the OPACS scales using Cronbach's alpha. The structure of each of the OPACS scales (practice and general opinion) was assessed using exploratory factor analyses, using the maximum likelihood method of extraction with varimax rotation. While the sample size was relatively small, Guadagnoli and Velicer determined that a sample size of 150 is adequate to produce a stable solution if factors comprise 10 or more items with loadings >0.4 (Guadagnoli & Velicer, 1988).

Exploratory factor analysis was used to provide insight into the structure of the OPACS and is a method that explores the data set and tests predictions in an attempt to uncover complex patterns (Child, 2006). It enables identification of the underlying factor structure.

Three separate factor analyses were performed to maximize the number of participants per included item in each analysis. It was hypothesized that all 36 items representing behaviours (section A, practice) would form a single factor. Further, it was hypothesized that the 29 items of the attitude subscale and the 21 items of the knowledge subscale would similarly form single factors.

Pearson's correlation coefficients were calculated between the three OPACS subscales and then between the OPACS subscales and total scores on the PFAQ and CES to assess the construct validity of the OPACS scales. It was expected that correlations between the three OPACS subscales would be medium in size to reflect overlapping but distinct concepts and that specific correlations between the OPACS and other measures (i.e., OPACS knowledge with PFAQ; OPACS attitude with CSE) would be high and negative, but other correlations between OPACS subscales and criterion measures would be low or medium in size.

## 3 RESULTS

### 3.1 Reliability

The OPACS had good internal reliability on both scales (*α* = 0.88 practice scale; *α* = 0.90 general opinion scale) when used with Australian BN students. When the general opinion scale was further divided into knowledge and attitude subscales, good internal reliability was again demonstrated (*α* = 0.82 for knowledge; *α* = 0.84 for attitude).

### 3.2 Exploratory factor analysis

Maximum likelihood factor analysis with varimax rotation was first conducted on the 36 items of the practice scale. The sampling adequacy for the factor analysis was verified by the Kaiser–Meyer–Olkin measure, KMO = 0.812 (values between 0.7–0.8 are good; Field, 2014). A one‐factor solution accounted for 23.0% of the variance in items. Only 13 items had a factor loading above 0.45 and communalities >0.2 and the goodness‐of‐fit test was significant (*χ* ^2^ = 1618.41, *p* < 0.000), suggesting poor fit.

The 50 items of the general opinion scale were divided into two subscales measuring attitude and knowledge. The sampling adequacy for the factor analysis of the 29 items of the attitude subscale was verified by the Kaiser‐Meyer‐Olkin measure, KMO = 0.817. A one‐factor solution accounted for 22.8% of the variance in items. Only 15 items had a factor loading above 0.45 and communalities >0.2 and the goodness‐of‐fit test was significant (*χ* ^2^ = 837.6, *p* < 0.000), suggesting poor fit.

The sampling adequacy of the 21 items of the knowledge subscale was verified by the Kaiser‐Meyer‐Olkin measure, KMO = 0.812 for the 21 items of the knowledge subscale. A one‐factor solution accounted for 25.6% of the variance in items. Only 12 items had a factor loading above 0.45 and communalities >0.2 and the goodness‐of‐fit test was significant (*χ* ^2^ = 390.7, *p* < 0.000), suggesting poor fit for this subscale also.

### 3.3 Criterion validity

Criterion validity was assessed using Pearson's correlation coefficients (Table 3). Strong correlations (all over 0.59) were observed between the OPACS subscales. There was no correlation between the PFAQ and OPACS practice and low negative correlations between the PFAQ and both the subscales of the OPACS general opinion (knowledge and attitude). Correlations between the CES and the OPACS subscales were low and negative (for practice and attitude) or non‐existent (for knowledge; Table 4).

**Table 3:** Correlations between OPACS subscales (Pearson's *r*)

	Practice	Attitude	Knowledge
Practice	—	0.662**	0.585**
Knowledge	0.585**	0.710**	—
Attitude	0.662**	—	0.710**

** Correlation is significant at the 0.01 level (2‐tailed).

**Table 4:** Correlations between OPACS subscales and external measures

	PFAQ	CES
Practice	−0.07	−0.23**
Knowledge	−0.19*	−0.16*
Attitude	−0.22**	−0.24**

* Correlation is significant at the 0.05 level (2‐tailed).

** Correlation is significant at the 0.01 level (2‐tailed).

## 4 DISCUSSION

Despite possessing satisfactory internal reliability, none of the OPACS subscales proved to reflect a single underlying factor and tests of validity indicated unsatisfactorily low correlations with criterion measures.

The current study found acceptable internal reliability for the three OPACS subscales, measured using Cronbach's alpha. However, a high alpha may result from including a large number of items, rather than because the items cohere meaningfully (Field, 2014). A factor analysis is usually recommended given well‐known limitations of coefficient alpha as a measure of unidimensionality or homogeneity in a sample of test items (Tavakol & Dennick, 2011).

Exploratory factor analyses indicated that the items of neither section A (practice) nor section B (attitude and knowledge) sample from distinct underlying constructs.

It was expected that correlations between the three subscales of the OPACS would be moderate in size, because each of the subscales should be predicted by, but not substantially overlap with, the other two. Higher correlations between the three subscales that might be expected on theoretical grounds were observed, suggesting that items from the three scales sample from a single underlying dimension.

Knowledge of ageing would be expected to have a low negative correlation with (poor) practice, a medium‐to‐high negative correlation with (poor) attitude and a high positive correlation with knowledge. The correlations between the OPACS practice and the criterion measure of knowledge were lower than expected and suggest the OPACS knowledge subscale is only minimally related to knowledge of ageing.

Similarly, a measure of positive attitudes towards caring for older patients would be expected to have a medium‐to‐high negative correlation with (poor) attitude and low negative correlations with (poor) practice and (low) knowledge. However, all correlations between the measure of caring self‐efficacy and the OPACS subscales were low (although higher than correlations between the criterion measure of knowledge and OPACS subscales). This poor result may be because of the complex nature of attitude (Dikken, Hoogerduijn, Lagerwey, et al., 2017) and that attitude towards older patients is not a one‐dimensional construct. Alternatively, it may be because some OPACS items are not good measures of attitudes towards older people. For example, it is difficult to see how the intention to involve younger patients in their hospital care is related to ageist attitudes, except when compared with the intention to involve older adults in their care.

Although originally described as a measure of knowledge, attitudes and practice of nurses regarding care of older hospitalized patients (Courtney et al., 2000; Malmgreen et al., 2009), more recent studies have largely concluded that revised versions of the OPACS are, at best, measures of attitudes only (Dikken, Hoogerduijn, Klaassen, et al., 2017; Dikken, Hoogerduijn, Lagerwey, et al., 2017; van Schelven et al., 2015). This study of the OPACS confirms these conclusions in the Australian context.

Given evidence that attitudes towards older patients are established very early in nurses’ professional lives, the decision was made to recruit a sample of student nurses for the current study, to explore the attitudes of nurses before they embark on their careers in acute care. This choice of sample contrasts with that of previous studies attempting to validate the OPACS that have employed samples of qualified nurses. It is possible that this difference in samples is responsible for differences in results between the current study and previous studies that have supported use of the OPACS (Courtney et al., 2000; Malmgreen et al., 2009). Arguably, student nurses’ attitudes are less coherent and well‐formulated than the attitudes of registered nurses. Future research could usefully explore changes in nurses’ attitudes towards older patients longitudinally, especially in the transition to becoming a professional nurse.

### 4.1 Limitations

Generalizability of the results of this study may be limited by use of a convenience sample from the same university and the study's relatively small sample size. However, a more diverse sample may have resulted in lower estimates of internal reliability of the subscales and even lower assessment of factor structure and construct validity.

A further limitation is the study's reliance on self‐report. Nurses’ self‐reports of their attitudes may not accurately predict their behaviours. Self‐report scales are notoriously poor measures of behaviours, creating the potential for the introduction of bias because of participant self‐selection and “faking good” in an attempt to conform to socially acceptable values (van de Mortel, 2008). For example, health professionals generally over‐estimate the extent to which they comply with hand washing (Jenner et al., 2006). Such bias could have led to inflated estimates of internal reliability and construct validity.

## 5 CONCLUSION

Worldwide, older people account for an increasing proportion of patients in hospital; for many, the experience has negative outcomes including loss of functional capacity. Because nurses’ knowledge and attitudes influence how and how well, care is provided (and to assess the effectiveness of any interventions to improve knowledge, attitudes and practice) having a means to measure these constructs is essential. Despite finding the OPACS has good internal reliability when trialed with Australian BN students, this study determined that the items of the OPACS do not sample from coherent underlying constructs as was expected, suggesting it is not a satisfactory measure of nurses’ knowledge, attitudes or practices regarding older hospitalized patients. However, analyses of construct validity indicated that the OPACS more closely resembles a measure of attitudes than knowledge or practice. The complex multi‐dimensional nature of attitudes towards older patients may contribute to the failure of the attitude items to load on a single factor. The current study confirms the findings of previous studies suggesting that the OPACS is a measure of attitudes only. Similarly, to these previous studies, we cannot recommend the use of OPACS in its current form.

## CONFLICT OF INTEREST

No conflict of interest has been declared by the author(s).
